# Superresolution mapping of energy landscape for single charge carriers in plastic semiconductors

**DOI:** 10.1038/s41467-018-06846-2

**Published:** 2018-10-17

**Authors:** Yifei Jiang, Jason McNeill

**Affiliations:** Department of Chemistry, Clemson University, Clemson, SC 29634 USA

## Abstract

The performance of conjugated polymer devices is largely dictated by charge transport processes. However, it is difficult to obtain a clear relationship between conjugated polymer structures and charge transport properties, due to the complexity of the structure and the dispersive nature of charge transport in conjugated polymers. Here, we develop a method to map the energy landscape for charge transport in conjugated polymers based on simultaneous, correlated charge carrier tracking and single-particle fluorescence spectroscopy. In nanoparticles of the conjugated polymer poly[9,9-dioctylfluorenyl-2,7-diyl)-*co*-1,4-benzo-{2,1′-3}-thiadiazole)], two dominant chain conformations were observed, a blue-emitting phase (*λ*_max_ = 550 nm) and a red-emitting phase (*λ*_max_ = 595 nm). Hole polarons were trapped within the red phase, only occasionally escaping into the blue phase. Polaron hopping between the red-emitting traps was observed, with transition time ranging from tens of milliseconds to several seconds. These results provide unprecedented nanoscale detail about charge transport at the single carrier level.

## Abstract (web summary)

To understand the complex nanoscale structure-property relationships in conjugated polymers for device applications, new methods for tracking charge transport are required. Here, the authors employ superresolution mapping to study the charge carrier dynamics in conjugated polymer nanoparticles.

## Introduction

Conjugated polymers exhibit many useful properties, including tunable electronic band gaps, and excellent flexibility and processability^1–3^. However, the structures of conjugated polymer devices are typically heterogeneous, due to the flexibility of conjugated polymer chains and weak inter-chain interactions^4–6^. The complexity of the structure results in complex or dispersive charge transport and limits the performance of conjugated polymer based devices^6–8^. Currently, there is considerable interest in obtaining a deeper understanding of the relationship between nanoscale structure and charge transport properties. Time-of-flight and voltage–current studies have provided useful information about charge transport in conjugated polymer films^9,10^. More recently, femtosecond pump-push spectroscopy has provided additional understanding of the early steps of charge separation^11,12^. However, as ensemble measurements, these methods involve averaging over large areas and many charge carriers. As such, questions remain regarding how local heterogeneity and the associated nanoscale energy landscape dictate the timescales and length scales of carrier motion, as well as the relationship between structure and carrier motion, in conjugated polymers. Single-molecule methods are uniquely suited to address heterogeneity and characterize complex, unsynchronized sequences of events^13,14^. Recently, we demonstrated superresolution tracking of single charge carriers in conjugated polymer nanoparticles, finding complex motion occurring over several timescales and length scales^15^. In this paper, we extend this approach by developing a novel method to map the nanoscale energy landscape for single charge carrier transport in conjugated polymers based on simultaneous, correlated superresolution charge carrier tracking and single-particle fluorescence spectroscopy.

Hole polarons are known to be efficient fluorescence quenchers in conjugated polymers^16–19^. When a hole polaron is present in a conjugated polymer nanoparticle (CPN) or an isolated conjugated polymer chain, it quenches the local emission, resulting in displacement of the fluorescence centroid and changes in the single-particle emission spectrum^16,20,21^. As we reported previously, the fluorescence centroid displacement can be used to indicate the location of a polaron inside a nanoparticle^15,21^. By subtracting a quenched (after polaron generation) spectrum from a unquenched (before polaron generation) spectrum, we obtain the spectrum of emission sites quenched by a hole polaron, at a given time. As a hole polaron moves inside a CPN, different parts of the nanoparticle are quenched, thus generating a nanoscale map of local emission characteristics inside a CPN.

We used this method to study nanoparticles of conjugated polymer poly[9,9-dioctylfluorenyl-2,7-diyl)-*co*-1,4-benzo-{2,1′-3}-thiadiazole)] (F8BT). We observe a two-tier energy landscape, with one feature characterized by a broad, featureless emission spectrum with *λ*_max_ around 550 nm, and the other characterized by a narrow, red-shifted emission spectrum showing clear vibronic structure with *λ*_max_ around 595 nm. Photo-generated hole polarons were quickly captured by the red-emitting phase within tens to hundreds of milliseconds. The hole polarons were highly trapped within the red-emitting regions, only occasionally escaping into the blue phase. When there are multiple nearby red-emitting trap sites, transitions between the traps were observed. The transition time was determined via change point analysis, and ranged from tens of milliseconds to several seconds. Overall, these results provide an unprecedented, single carrier level of detail about the complex charge transport processes of conjugated polymers, and improve our understanding of the relationship between nanostructure and charge transport properties.

## Results

### Single-particle imaging and polaron tracking

Nanoparticles of conjugated polymer F8BT were prepared using the nano-reprecipitation method described previously^22–25^. The freshly prepared CPNs were filtered using a cross-flow filtration column (pore size ~10 nm) to narrow the nanoparticle size distribution. After the filtration, an average particle size of 11.2 ± 3.7 nm was obtained from dynamic light scattering measurement (Supplementary Note 1 and Supplementary Fig. 1). The particles were dispersed on a glass coverslip and imaged with an inverted fluorescence microscope, for 200 s. A transmission grating was placed in front of the detector to disperse the single-particle fluorescence. Approximately Gaussian-shaped point spread functions (PSFs) were observed at the zeroth order fluorescence spot and long stripes were observed at the first-order diffracted fluorescence. Single-particle fluorescence spectra were determined from the first-order diffracted fluorescence by summing each stripe along the *Y* axis (Supplementary Methods and Supplementary Fig. 2). At the zeroth order (not diffracted) fluorescence spot, the PSFs of CPNs were fitted to a 2D Gaussian function to determine the fluorescence centroid. Under 100 W cm^−2^ excitation power density, 1–3 × 10^3^ photons were detected at the zeroth order spot, per particle, per 20 ms exposure, resulting in a localization precision of 2.5–3.8 nm per frame (Supplementary Note 2). When a quencher (hole polaron) was generated, due to the fact that fewer photons were detected, the tracking uncertainty slightly increased. Depending on the size of the CPN, generation of a hole polaron typically results in a quenching depth of 10–40% (Supplementary Note 3 and Supplementary Fig. 3). In most of our experimental conditions, the tracking uncertainty is shot-noise limited. During the intensity off state, the localization uncertainty increased by 5–30%, compared to the localization uncertainty during the intensity on state. The abrupt centroid position jumps observed during the intensity off state are above the expected localization uncertainty, which indicates that the fluorescence centroid movements were caused by internal dynamics of CPNs. As discussed previously, a hole polaron can induce a dark spot inside a CPN, which moves with the polaron and results in the fluctuations in the fluorescence centroid^15,20,21^. To determine the position of the hole polaron, we employed the relationship we previously developed ^21^,

1 $$\delta x_{\mathrm{f}} \approx - x_{\mathrm{p}}Q_{\mathrm{p}},$$

where δ*x*_f_ is the displacement of the fluorescent centroid caused by polaron, *x*_p_ is the position of the polaron relative to the center of the particle, and *Q*_p_ is the quenching efficiency of a single polaron, which is estimated from the quenching depth of the blinking events in the single-particle fluorescence intensity trajectories.

### Single-particle spectrum analysis

**Fig. 1:**
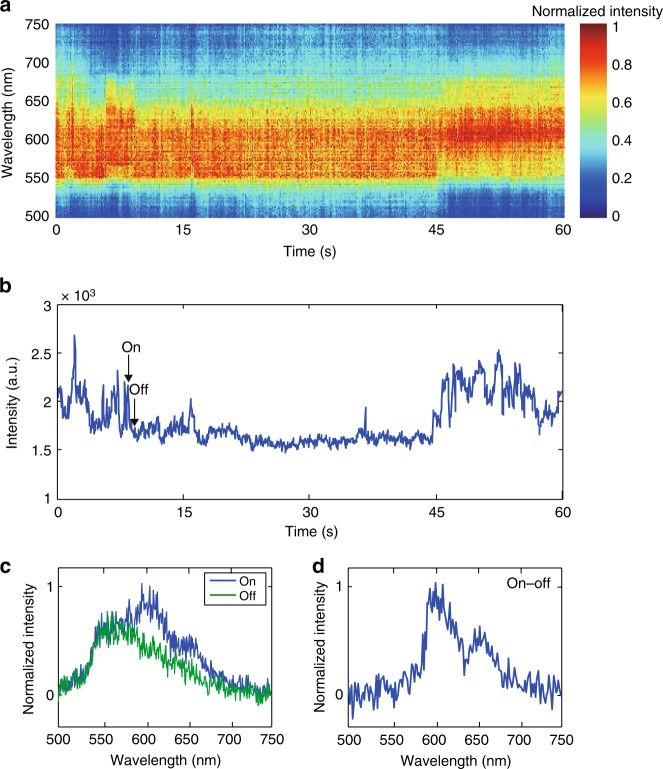
Single-particle spectrum and fluorescence intensity trajectory of a F8BT CPN. **a** Stacked single-particle fluorescence spectra showing changes over time. The shifts in the spectra can be correlated with blinking events in the corresponding fluorescence intensity trajectory shown in plot (**b**). **b** The corresponding fluorescence intensity trajectory exhibiting pronounced blinking behavior. **c**, **d** The spectra of **c** on and off states, **d** on–off state. For the on–off spectrum plot, two-pixel binning along the *X* axis (wavelength) was performed to reduce noise

In the single-particle fluorescence intensity trajectories, we observed abrupt fluorescence intensity jumps likely caused by reversible generation and recombination of polarons (see Supplementary Note 4 for likely hole polaron generation mechanisms). The single-particle fluorescence spectra of CPNs also exhibit significant changes over time. In Fig. 1a, we stacked a series of single-particle fluorescence spectra together to illustrate the shifts in the emission wavelength. The corresponding fluorescence intensity trajectory is shown in Fig. 1b. The shifts in the spectra are highly correlated with the blinking events—CPNs typically exhibit red emission during the on state (higher intensity) and blue emission during the off state (lower intensity). If we assume that the shifts are due to selective quenching of some emitting species, then by subtracting an off state spectrum from the adjacent on state spectrum, we obtain the spectrum of the emission sites quenched by the hole polaron, at a given time^16^. The difference spectra are typically red-shifted with clear vibronic structure (Fig. 1d), likely indicating that the emission comes from a few low-energy sites.

It should be noted that there are a small fraction of the CPNs (~5%) that exhibited only blue emission with no red-emitting characteristic. The fluorescence intensity trajectories of these CPNs show rolling fluctuations rather than discrete jumps and the shape of single-particle spectra remains unchanged overtime (Supplementary Fig. 4 and Supplementary Fig. 5). Analysis of the intensity trajectories of dozens of CPNs revealed that polarons generated in the red-emitting regions resulted in significantly higher quenching depths than polarons generated in the blue-emitting regions (Supplementary Note 3 and Supplementary Fig. 3). The narrow emission spectrum and efficient exciton quenching by hole polarons observed in the red-emitting phase are consistent with the previous observation of low energy emission site/energy funnels in single-molecule study of conjugated polymers^13,16^. The structural origin of the low-energy sites could be areas with chain–chain contact (but without precise spatial alignment of molecular orbitals required for an excimer) or polymer chain segments with extended conjugation length^16,26,27^. A previous single-molecule study showed that long-chain F8BT molecules with collapsed conformation tend to have more red-emitting sites, likely due to the fact that longer collapsed polymer chains tend to have more areas with chain–chain contact^28^. Previous studies of F8BT films have also provided insights about structural origin of the low energy emission. It was previously observed that annealing of the F8BT films reduced red-emitting characteristics and significantly lowered charge carrier mobilities^29^. Modeling shows that, before annealing, the BT units are stacked face to face, resulting in efficient inter-chain charge and exciton migration. After annealing, the F8BT chains adopt lower energy packing, in which the BT units in one polymer chain are adjacent to F8 units in a neighboring chain (alternating structure). The alternating structure results in less efficient inter-chain energy transfer and reduces red-emitting characteristics in the emission spectrum. These results are consistent with our observation that exciton migration to quenchers in the blue-emitting phase is less efficient. The featureless, high energy emission spectra observed are likely due to a large number of localized emission sites. Of course, the relationship between structure and spectroscopic features is complex, and there are other possible structures that can result in the blue-shifted and red-shifted features, such as H-aggregate or J-aggregate species, which cannot be ruled out by current measurement.

**Fig. 2:**
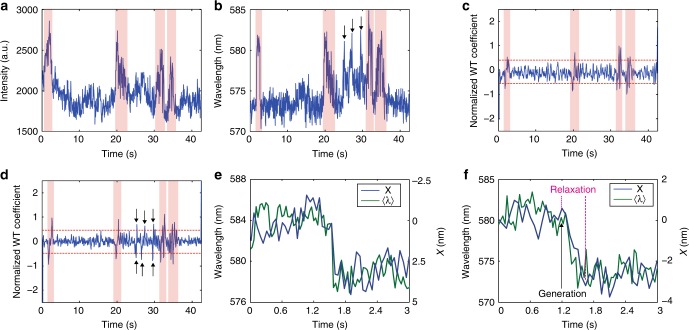
Change point analysis of single-particle intensity and spectrum centroid trajectories. **a** A single-particle fluorescence intensity trajectory showing two-level blinking behavior. **b** The corresponding spectrum centroid trajectory. **c**, **d** Haar wavelet transform coefficient trajectories of the intensity trajectory (**c**) and the spectrum centroid trajectory (**d**). The peaks in the wavelet transform coefficient trajectories indicate change points in the original trajectories (thresholds to differentiate local maximum from noise are shown by the red dashed lines). The red bands indicate the spectrum shifts that are correlated with the blinking events. The black arrows indicate the spectrum change points that are uncorrelated with the blinking dynamics. **e** The X position trajectory of the fluorescence centroid of a CPN (blue) and the corresponding spectrum centroid trajectory (green), showing a discrete jump during the polaron generation process. **f** The X position trajectory of the fluorescence centroid of a CPN (blue) and the corresponding spectrum centroid trajectory (green), showing continuous drift during the polaron generation process

To quantify the spectrum shift, we defined the spectrum centroid as follows:

2 $$\lambda = \frac{{\mathop {\int }\nolimits_{500}^{750} \lambda i(\lambda ){\mathrm d}\lambda }}{{\mathop {\int }\nolimits_{500}^{750} i(\lambda){\mathrm d}\lambda }},$$

where *λ* is emission wavelength and *i*(*λ*) is the emission intensity as a function of wavelength. We used the Haar wavelet transform to detect change points in the fluorescence intensity and spectrum centroid trajectories (Supplementary Methods and Supplementary Fig. 6). It was observed that most of the spectrum shifts occur simultaneously with fluorescence blinking events (highlighted by the red bands in Fig. 2a–d), consistent with polaron generation and recombination dynamics. However, sometimes, the spectrum centroid trajectories can also exhibit abrupt changes during the intensity off state (shown by the black arrows in Fig. 2b, d), which cannot be explained by polaron generation/recombination dynamics. A possible explanation is polaron hopping dynamics. There is evidence that hole polarons can move between a few sites^15^. As a hole polaron moves inside a CPN, different areas (with different emission characteristics) are quenched, which could results in changes in the spectrum centroid. We will discuss this phenomenon in detail below.

We used the single-particle localization method discussed above to indicate the location of the polaron over time. In some trajectories, when a hole polaron is generated, we observed discrete jumps in the centroid position of the fluorescence spot and the spectrum centroid (Fig. 2e), consistent with a quencher being generated on a red-emitting site. Meanwhile, in other cases, we observed that, when a hole polaron is generated, the fluorescence centroid position either exhibits a continuous drift or a multiple step jump, finally stabilizing after tens to hundreds of milliseconds (Fig. 2f). At the same time, the spectrum centroid also exhibits continuous changes or complex fluctuations. After the fluorescence stabilizes, the red shoulder of the single-particle spectrum is quenched, likely indicating that the hole polaron is relaxed to (rather than initially generated within) a red-emitting region (Supplementary Fig. 7). Once a polaron is captured by a red-emitting region, it becomes trapped, i.e., the fluorescence centroid does not appear to move for a time. It is also observed that in some cases, for a given particle, during different generation/recombination cycles, the centroid tends to drift to the same location, indicating that the hole polaron tends to be captured by the same trap site (Supplementary Fig. 8). Charge trapping by red-emitting regions can be explained by the fact that polarons in ordered domains are stabilized by delocalizing along and (or) between polymer backbones, thus possessing lower energies than polarons in glassy regions^30^. Also, in a semi-crystalline system, it is statistically favorable for charge carriers at the grain boundary to hop back to the ordered areas^4^. Another possibility is that the red-emitting hole polaron trap sites could result from chemical or structural defects.

### Number of hole polaron trap sites

**Fig. 3:**
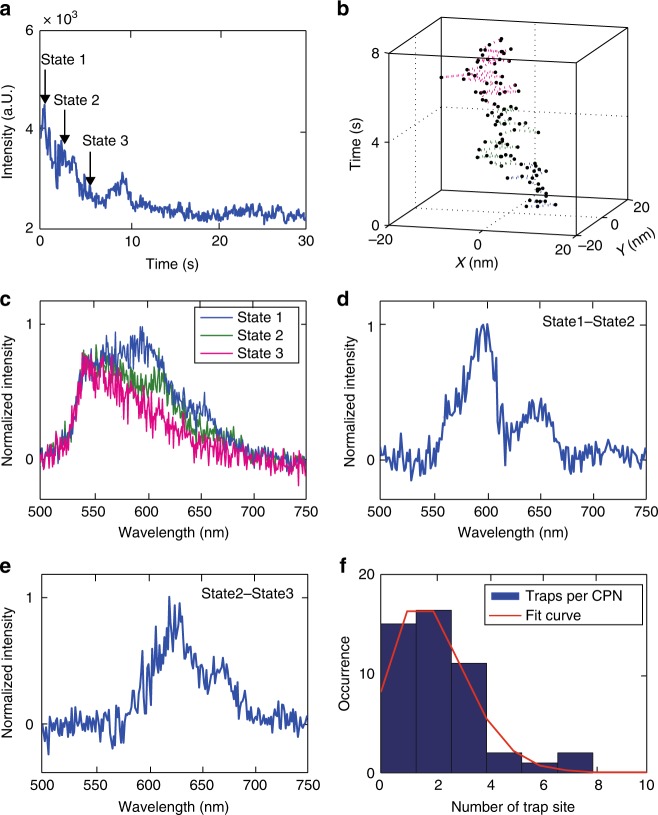
Intensity decays caused by multiple hole polaron generation events. **a** A fluorescence intensity trajectory showing stepwise decay at early time. **b** The corresponding centroid position trajectory during the fluorescence decay, acquired at 50 Hz framerate. Different color indicates different state in the intensity decay. For plotting, every 5 points in the position trajectory were binned together to reduce noise. **c** Single-particle fluorescence spectra at different states of the decay. **d** State1–state2 spectrum. **e** State2–state3 spectrum. For plots **d** and **e**, every two pixels along the *X* axis (wavelength) were binned together to reduce noise. **f** The distribution of number of red-emitting sites/charge carrier traps per CPN, fitted to a Poisson distribution with *λ*=3

In some particles, typically at higher excitation intensities, a rapid fluorescence intensity decay within the first 10–20 s can be observed (Fig. 3a), which has been previously attributed to growth of polaron population to a steady state value^31^. For these particles, as the polaron population approaches equilibrium, stepwise shifts in the fluorescence centroid were observed (Fig. 3b), likely indicating each successive polaron occupying a different site or trap. To examine this process, we performed a multi-step spectrum subtraction analysis to obtain the spectrum of each individual charge carrier trap. An example is given in Fig. 3. The decay in the fluorescence intensity and shifts in the fluorescence centroid in Fig. 3a, b can be separated into three states. The spectrum of each state was determined after the fluorescence centroid stabilized. The spectrum of the state 2 was subtracted from the spectrum of the state 1 to recover the spectrum of the first trap site. We then subtracted the spectrum of the state 3 from the spectrum of the state 2 to recover the spectrum of the second trap. In this case, the spectra of the two traps show considerable difference in *λ*_max_. However, both spectra are sharp and red-shifted compared to the spectrum of the final state (state 3), indicating that, at equilibrium, the low-energy sites inside the particle were occupied by hole polarons. Based on this phenomenon, we estimated the number of low energy sites per particle from the number of steps in the initial fluorescence decay. It is found that the number of red-emitting sites per particle is consistent with a Poisson distribution with an average trap density of roughly three traps per particle.

### Polaron hopping dynamics

**Fig. 4:**
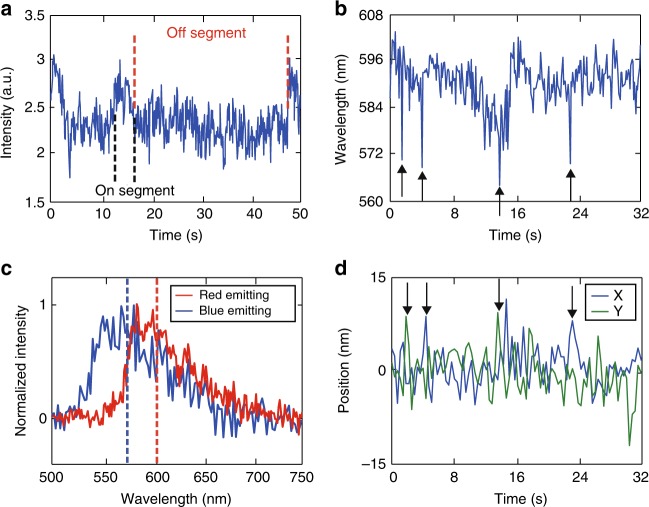
Polaron hopping between the red and the blue phase. **a** A fluorescence intensity trajectory showing two-level blinking. **b** The spectrum centroid trajectory determined from the off segment in plot **a**, showing two-level fluctuations. **c** Typical difference spectra, showing shifts in the spectrum centroid (indicated by the dashed lines). For this plot, every two pixels along the *X* axis (wavelength) were binned together to reduce noise. **d** The *X* and *Y* polaron position trajectory obtained during the lower intensity segment in plot a, originally acquired at 50 Hz framerate. For plotting, every 10 points were binned together to reduce noise, both *X* and *Y* trajectories were moved to center around 0. Polaron hopping events that occur together with the downward spikes in the spectrum centroid are highlighted by the arrows

As briefly discussed earlier, while hole polarons are trapped most of the time, occasionally they escape from the trap sites, as indicated by abrupt jumps in the fluorescence centroid with simultaneous pronounced shifts in the fluorescence spectrum. In order to determine which part of the CPN was quenched by a hole polaron over time, we performed the following analysis. We selectively studied CPNs exhibiting two-level intensity fluctuations (Fig. 4a), as the motion of multiple polarons could complicate the analysis. When there is no polaron present in the CPN, the unquenched spectrum was determined by averaging over all the spectra obtained during a fluorescence on event. In the next off segment (i.e., upon formation of a polaron), we subtract the quenched spectrum frame by frame from the unquenched spectrum to recover the spectra of the emission sites quenched by the hole polaron over time (subtracted spectra). To track the location of polaron over time, the centroid position trajectory obtained during an intensity off segment was converted to the polaron position trajectory using the relationship in Eq. (1). In Fig. 4b, we plotted the spectrum centroid trajectory of the subtracted spectra determined from the off segment in Fig. 4a. According to Fig. 4b, it is evident that the complex energy landscape for charge transport in a CPN can be approximately simplified to a two-tier model consisting of a blue-emitting conformation (*λ*<570 nm) and a red-emitting conformation (*λ*>590 nm). It is observed that the spectrum centroid fluctuates above 590 nm for most of the time, consistent with highly trapped polaron within the red-emitting phase. However, steep, short-lived decreases of the spectrum centroid can be observed occasionally (highlighted by the arrows in Fig. 4b), indicating that the hole polaron occasionally enters blue-emitting areas. The downward spikes in the spectrum centroid typically occur concurrently with polaron hopping events, indicated by sudden jumps in the polaron position trajectory (Fig. 4d). However, not all of the polaron hopping events exhibit a simultaneous spectrum centroid shift, likely due to hopping between sites with similar emission characteristics.

### Superresolution mapping

**Fig. 5:**
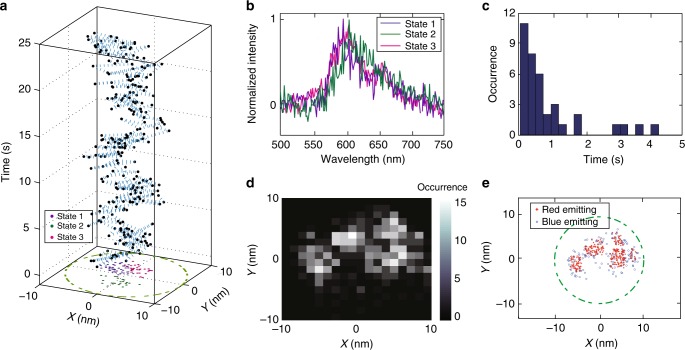
Superresolution mapping of the energy landscape inside a CPN. **a** The polaron position trajectory obtained during an intensity off segment, at 50 Hz framerate, showing a total of 3 distinguishable states. For this plot, every 5 points in the position trajectory were binned together to reduce noise. **b** The spectra of the three states shown in plot **a**. **c** Polaron hopping time histogram, determined from the change point analysis. **d** Polaron position histogram constructed from multiple polaron hopping trajectories and **e** the corresponding 2D scatter plot. The red and blue dots indicate the areas that exhibit red and blue emission, respectively

When multiple nearby trap sites are present, repeated polaron hopping between traps can be observed. A typical example is given in Fig. 5 (see Supplementary Note 7 and Supplementary Fig. 10 for additional polaron hopping trajectories). This polaron position trajectory exhibits abrupt jumps (Fig. 5a) with three distinguishable traps, based on clustering observed in the 2D polaron position scatter plot (Fig. 5b subplot). The spectrum of each trap site was determined using the subtraction method discussed above. While there are some differences in the shapes of the spectra (Fig. 5b), all spectra exhibit *λ*>590 nm and are consistent with the characteristics of low-energy sites. The transition times between the trap states were determined using change point analysis based on wavelet transform (Supplementary Methods). The transition times obtained from multiple polaron hopping trajectories were used to construct a polaron hopping time histogram. The histogram exhibits a long tail (Fig. 5c), which can be approximately fit to a power law or a stretched-exponential function (Supplementary Fig. 9), consistent with the power law waiting time distribution often featured in models of charge transport in disordered materials^32^. In order to obtain a nanoscale map of local emission characteristics inside a particle, we analyzed many polaron generation/recombination events of a single particle. The polaron position trajectories obtained during all the intensity off states were used to construct a 2D polaron position histogram. It is observed that the polaron spent most of the time at a few areas (Fig. 5d), likely indicating the location of the trap sites. In the corresponding spectrum centroid trajectories, we observed two-level fluctuations, consistent with the two-tier energy landscape discussed above. To illustrate local emission characteristics inside the particle, we constructed a 2D scatter plot from the polaron position trajectories, the red dots indicate that the area exhibits red emission and the blue dots indicate that the region exhibits blue emission, respectively (Fig. 5e). It is observed that the polaron has most occurrences in the red-emitting areas, which is additional evidence that the red-emitting sites act as charge carrier traps. In this plot, we observed that there are approximately 4 traps inside this particle, which is consistent with the trap density determined from the analysis of the fluorescence intensity decay.

## Discussion

In summary, we demonstrated superresolution single charge carrier tracking with simultaneous, correlated single-particle spectrum analysis, as a method for mapping the nanoscale energy landscape of charge carriers in particles of conjugated polymer. A hole polaron can efficiently quench the local emission in a particle, resulting in displacement of the fluorescence centroid and changes in the emission spectrum. Using a spectrum differencing scheme, we obtain the spectra of emission sites quenched by a hole polaron, at a given time. As a polaron travels inside a CPN, there are site-to-site variations in the difference spectra, which reveal nanometer-scale site-to-site variations in the emission spectrum of the polymer in the vicinity of trap sites. We calculated the spectrum centroid of the difference spectrum at each time step and observed two-level fluctuations over time, likely indicating that there is a two-tier energy landscape for charge transport in CPNs. The spectra can be roughly grouped into two types, a broad, featureless emission spectrum with *λ*_max_ around 550 nm and a narrow, red-shifted emission spectrum with *λ*_max_ around 595 nm with clear vibronic structure. Photo-generated hole polarons were quickly captured by the red-emitting areas within tens to hundreds of milliseconds. It was observed that the hole polarons were highly trapped within the red phase, only occasionally escaped into the blue phase. Polaron hopping between adjacent red-emitting traps were observed, with transition time ranges from tens of milliseconds to several seconds. It should be noted that hole polaron motion along the *Z* direction was not resolved by the fluorescence centroid measurement. We have observed spectrum centroid shifts that were uncorrelated with changes in the fluorescence centroid position, likely indicating polaron movements along the *Z* direction. However, we cannot distinguish two sites along *Z* direction that have similar emission characteristics. As a result, it is possible that we have overlooked some hopping events occurring primarily along the *Z* direction. However, since the particles are roughly spherical in this size range and chain-packing in CPNs is more or less random (i.e., no net average direction), it is likely that most of the phenomena observed along the *X* and *Y* directions hold true for the *Z* direction as well. The method developed here can simultaneously track the motion of individual charge carriers and provide information about the nanostructure in conjugated polymers. The results provide an unprecedented level of detail about dispersive charge transport on a complex energy landscape and improve our knowledge about the relationship between nanostructure and charge transport properties.

## Methods

### Nanoparticle preparation and characterization

Poly[(9,9-dioctylfluorenyl-2,7-diyl)-*co*-(1,4-benzo-{2,1′,3}-thiadiazole)] (F8BT, MW 10,000, polydispersity 1.7) was purchased from ADS Dyes, Inc. (Quebec, Canada). Tetrahydrofuran (THF, HPLC grade, 99.9%) and (3-Aminopropyl) trimethoxysilane (APS) were purchased from Sigma-Aldrich (Milwaukee, WI). All chemicals were used as received without further purification. F8BT nanoparticles were prepared by nano-reprecipitation method reported previously, described as follows^23,24,33^. Conjugated polymer F8BT was dissolved in THF and diluted to 20 ppm. A volume of 2 mL of the THF solution were injected into 8 mL of water under mild sonication. The mixed solution was then placed under partial vacuum to remove THF solvent. After evaporation, the sample was filtered through 100 nm polyvinylidene fluoride (PVDF) membrane (Millipore) to remove aggregates. Cross-flow filtration was performed with a porous polystyrene (PS) column (SpectrumLabs) to remove large particles. According to the absorption spectrum, <10% of the polymer was removed by filtering through PVDF membrane, indicating that most of the polymer formed nanoparticles. The nanoparticle size distribution was determined by atomic force microscopy (AFM) and dynamic light scattering (DLS). To prepare AFM samples, coverslips functionalized with APS were submerged under diluted CPNs suspension for 40 min. The coverslip topography was imaged with a multimode AFM (Ambios, Q250) in AC mode. The conditions employed for AFM scans were scan area of 5 μm, 500 lines per scan, and a scan rate of 0.5 Hz. The DLS measurements were conducted using a Nanobrook Omni (Brookhaven Instruments Corporation, NY) with BIC Particle Solutions Software (V 3.1). The DLS measurements were performed under the condition of 25 °C, count rate of 500 kcounts per second, scattering angle of 90°, and acquisition time of 180 s. PS nanospheres (Thermo Fisher, 24 nm) were used as a size standard. NNLS fitting was employed to determine particle size distributions. UV−vis absorbance and fluorescence spectra were collected using a Shimadzu UV-2101 PC scanning spectrophotometer and a commercial fluorometer (Quantamaster, PTI, Inc.), respectively.

### Single-particle imaging

Single-particle imaging was performed using a custom wide-field epifluorescence microscope, described as follows. A 445 nm laser diode (Thorlabs, LP450) is used as the excitation source. The excitation light is stabilized by passing through a liquid crystal noise eater (Thorlabs, NEL01). Then, the laser is guided to the rear epi port of an inverted fluorescence microscope (Olympus IX-71) using an optical fiber. In the microscope, the excitation light is reflected by a 500 nm long-pass dichroic (Chroma 500 DCLP) to a high numerical aperture objective (Olympus Ach, ×100, 1.25 NA, Oil). A Gaussian laser profile with full-width half maximum (FWHM) of ∼5 μm was observed at the sample plane. According to the laser input power, the width of the excitation spot and the transmission of the objective, we estimated that the typical excitation power density at the center of the laser spot is ~100 W cm^−2^. Nanoparticles of F8BT were dispersed on an APS functionalized glass coverslip using the same method for preparing AFM sample. A translation stage (Thorlabs, XYT-1) was used to position the sample. The CPNs were kept under N_2_ atmosphere and imaged for 200 s, using a framerate of 10 or 50 Hz. The fluorescence from the F8BT CPNs was collected by the objective lens, passed through a 500 nm long-pass filter and then focused onto a sCMOS camera (Neo sCMOS, Andor). The detector settings were 11 bits per pixel, gain setting of 0.6, rolling shutter mode. From the analysis of photon counting noise for a flat field, we determined the experimental gain factor to be 0.63 electrons per count. A pixel pitch of 65 nm per pixel was determined from imaging a TEM calibration grid. The fluorescence detection efficiency of the microscope is ~3%, determined by imaging nile red loaded polystyrene beads (Thermo Fisher) as standards. For measurement of single-particle fluorescence spectra, a transmission grating with 300 grooves per mm (Thorlabs, GT25-03) was placed between the 500 nm long-pass filter and the sCMOS camera to disperse fluorescence emission from CPNs. The dispersion factor is 0.7 nm per pixel, which is determined using a 540 ± 10 nm band pass filter (see SI for more information). Based on the single-particle fluorescence spot width (PSF) of roughly 4–5 pixels (FWHM), which roughly corresponds to the slit width of the spectrometer, the spectroscopic resolution is roughly 3.5 nm.

### Tracking of single hole polarons in CPNs

Tracking single hole polarons in CPNs was performed using a method we developed previously, described as follows^21^. Due to a hole polaron quenching the local fluorescence, the fluorescence centroid of CPNs can be used to indicate the position of the quencher. We first sum over all the frames and find the brightest pixel in the summed image. Based on the experimental signal to noise level, 5–10% of the intensity of the brightest pixel was used as a threshold to distinguish CPNs from the background noise. For pixels above the threshold, their intensities were compared to the neighboring pixels to roughly locate the central pixel of the fluorescence spot of each CPN. Based on the rough locations of CPNs, a 2D Gaussian function was fit to the fluorescence spot of each CPN, frame by frame. Typically 11 × 11 pixels were used for the fitting (5 pixels on each side of the central pixel). We checked the FWHM obtained from the fitting (~270 nm) to make sure it is not from multiple CPNs that are close to each other. In general, there are typically a few percent of the CPNs showing minimal photoblinking, consistent with large particles or aggregates observed in AFM measurement. It was observed that the non-blinking particles or aggregates in the image exhibit highly correlated slow movement in the centroid position, due to drift and vibrations from the environment. For each frame, the non-blinking particles were used as markers to measure at each frame how far the sample traveled relative to the previous frame. The frame-by-frame marker displacement was then subtracted from the position trajectories of the particles of interest in order to correct for vibration and drift.

## Electronic supplementary material

Supplementary Information

## Data Availability

The data sets generated during the current study are available from the corresponding authors on reasonable request.
