# Supplementary material for: Superresolution mapping of energy landscape for single charge carriers in plastic semiconductors
Source: Nat Commun. 2018 Oct 17;9:4314. doi: 10.1038/s41467-018-06846-2 (PMC6193038; doi:10.1038/s41467-018-06846-2)
Supplement: Supplementary file 1 — Supplementary Information [file 41467_2018_6846_MOESM1_ESM.pdf]

Supplementary Information

For

Superresolution Mapping of Energy Landscape for Single Charge Carriers in Plastic Semiconductors

Yifei Jiang et al.

Supplementary Figures

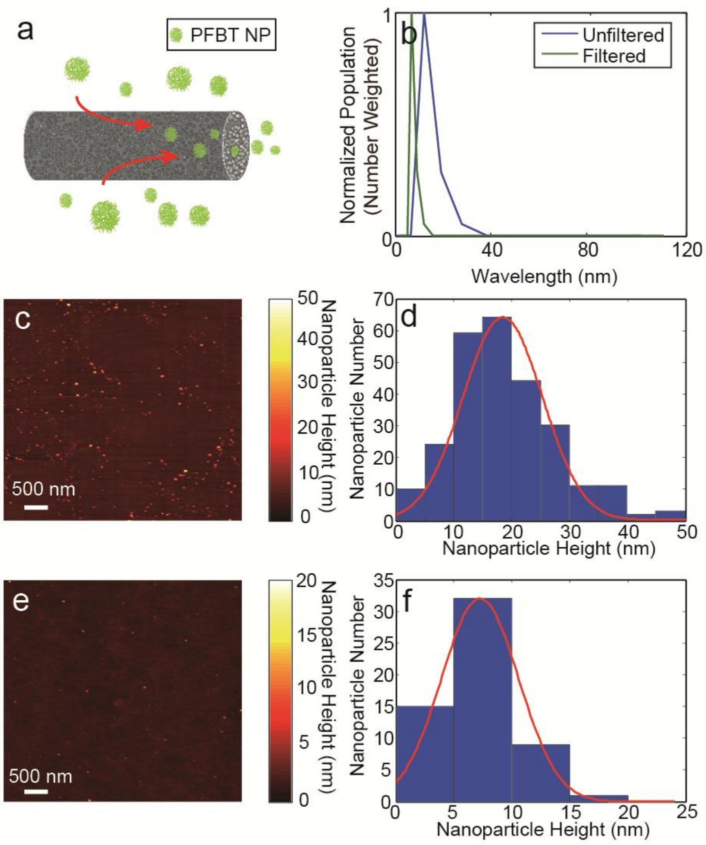

Supplementary Figure 1. Particle size distributions determined before and after cross-flow filtration. (a) Illustration of the cross-flow filtration process. (b) Number weighted nanoparticle size distributions before and after cross-flow filtration, determined from dynamic light scattering (DLS) measurement. (c) A representative atomic force microscopy (AFM) image of poly[9,9-dioctylfluorenyl-2,7-diyl)-*co*-1,4-benzo-{2,1′-3}-thiadiazole)] (F8BT) nanoparticles before cross-flow filtration. (d) The corresponding particle height distribution determined from plot (c). (e) A representative AFM image of F8BT nanoparticles after cross-flow filtration. (f) The corresponding particle height distribution determined from plot (e).

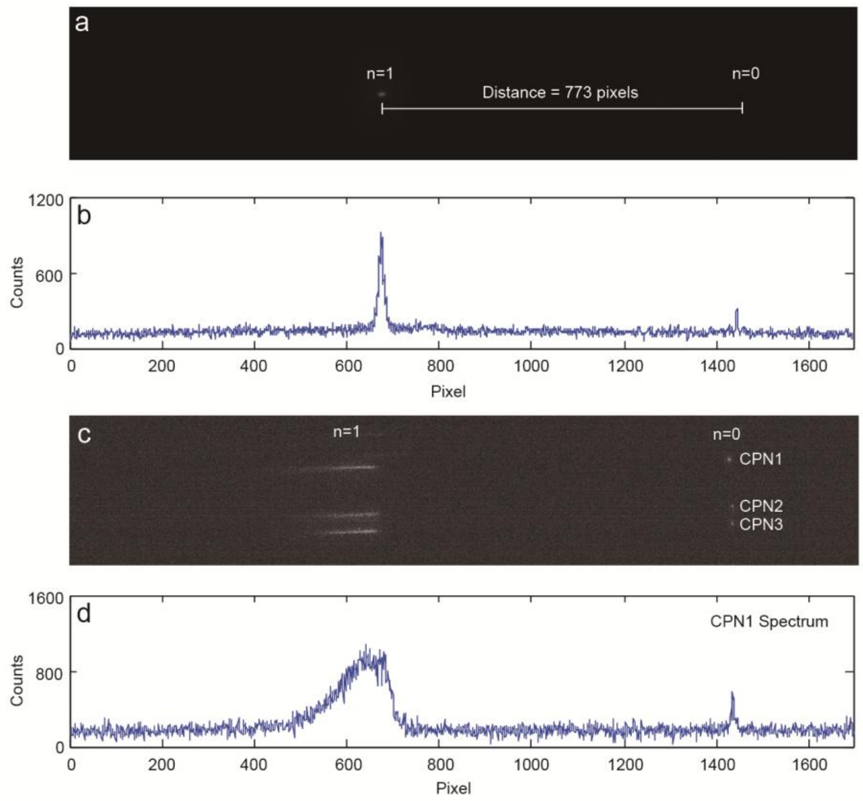

Supplementary Figure 2. Microscopy images of the zeroth and the first order fluorescence spots. (a) Fluorescence microscopy image of a conjugated polymer nanoparticle (CPN), passed through a 540±10 nm band-pass filter. The distance between the n = 0 and n = 1 spot is highlighted by the white line. (b)The profile of the n = 0 and n = 1 spots in figure (a), along X axis. (c) Fluorescence microscopy image of 3 CPNs, passed through a 500 nm long-pass filter. (b) The profile of the n = 0 and n = 1 spots of CPN1, along X axis.

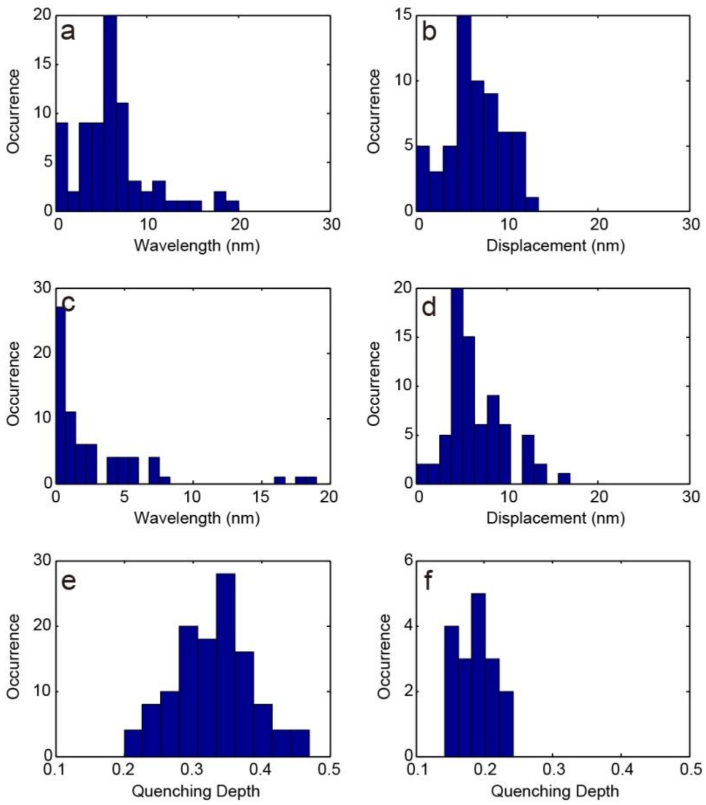

Supplementary Figure 3. Histograms of single particle statistics. (a)(b) Histograms of (a) spectral drift and (b) hole-polaron displacement associated with emission on/off transitions. (c)(d) Histograms of (c) spectral drift and (d) hole-polaron displacement associated with polaron hopping events. (e)(f) Histograms of hole polaron quenching depths in (e) mixed phase CPNs and (f) blue phase CPNs.

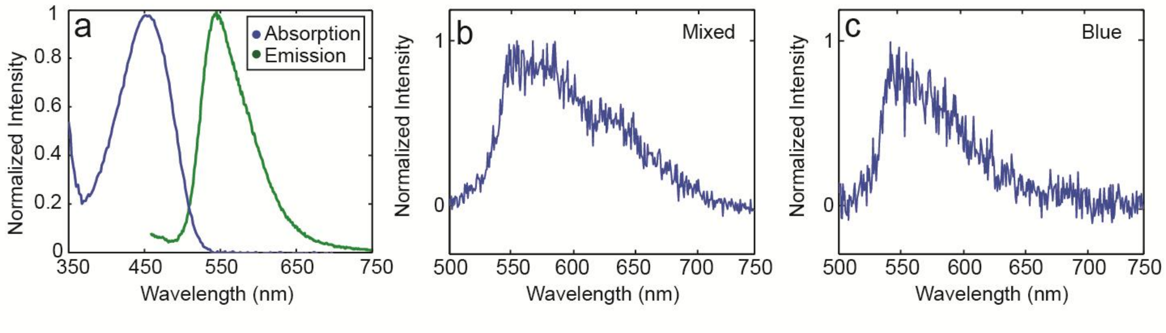

Supplementary Figure 4. Fluorescence spectra of bulk and single F8BT CPNs. (a) Absorption (blue) and fluorescence (green) spectra of bulk F8BT CPNs suspension. (b) Single particle fluorescence spectrum of mixed phase F8BT CPNs. (c) Single particle fluorescence spectrum of blue phase F8BT CPNs.

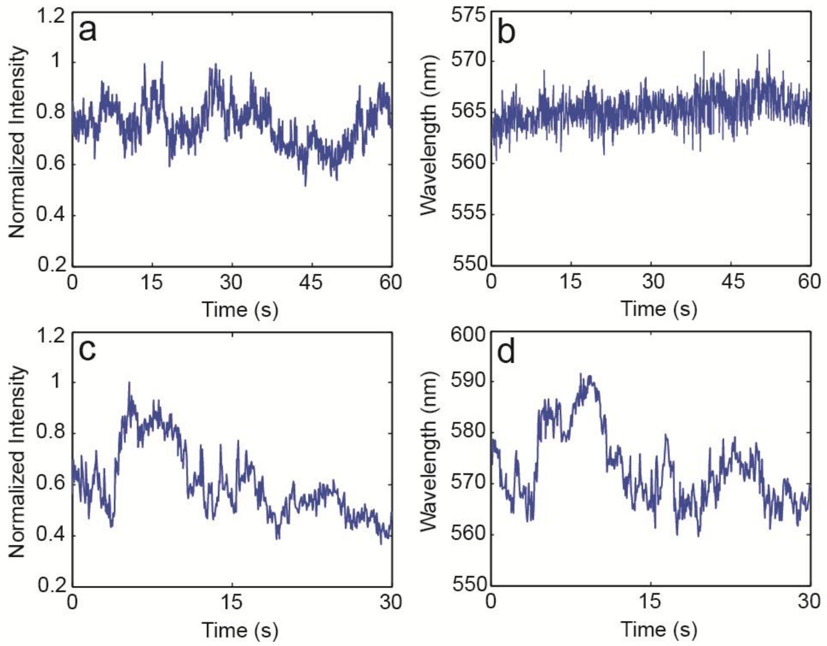

Supplementary Figure 5. Fluorescence intensity and spectrum centroid trajectories of blue and mixed phase CPNs. (a) The fluorescence intensity trajectory and (b) the corresponding spectrum centroid trajectory of a blue phase CPN. (c) The fluorescence intensity trajectory and (d) the corresponding spectrum centroid trajectory of a mixed phase CPN.

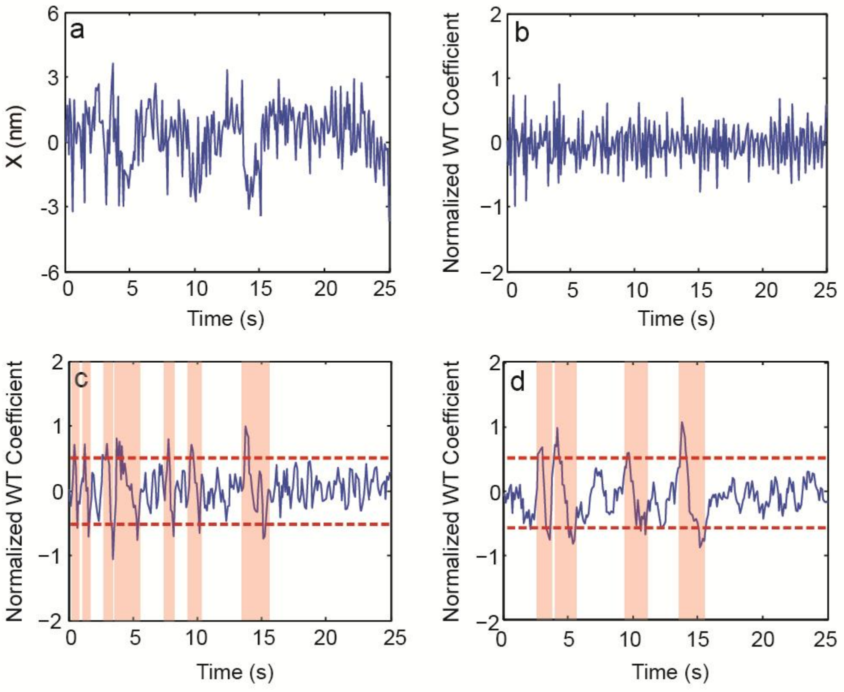

Supplementary Figure 6. Detection of change points in a polaron position trajectory using Haar wavelet transform. (a) A polaron position trajectory showing two-state hopping behavior. (b) The corresponding

wavelet transform coefficients at scale of 2. (c) The corresponding wavelet transform coefficients at scale of 6. (d) The corresponding wavelet transform coefficients at scale of 16. The threshold (red dashed lines) was calculated from median absolute deviation of the wavelet transform coefficients at scale 2. The identified transition events are highlighted by the red bands.

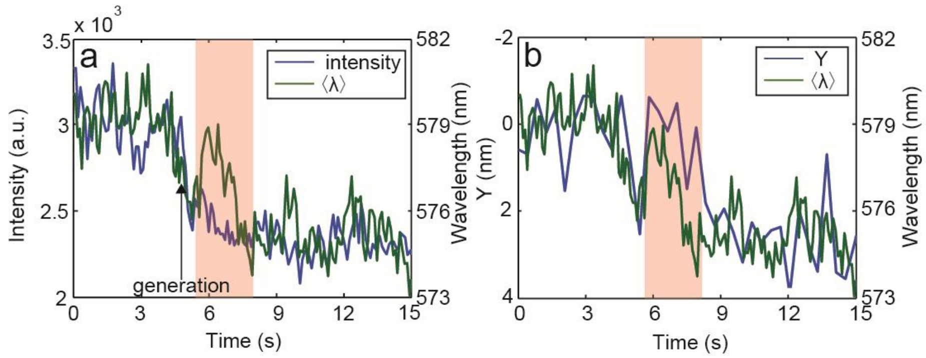

Supplementary Figure 7. Changes in fluorescence intensity, spectrum centroid and fluorescence centroid during a polaron relaxation process. (a) The fluorescence intensity (blue) and the spectrum centroid (green) trajectories of a CPN. A polaron generation event is indicated by the black arrow. During the relaxation process, the polaron briefly entered a blue-emitting area, which is highlighted by the red band. (b) The corresponding fluorescence centroid position along Y axis (blue) and the spectrum centroid (green) during the polaron relaxation process.

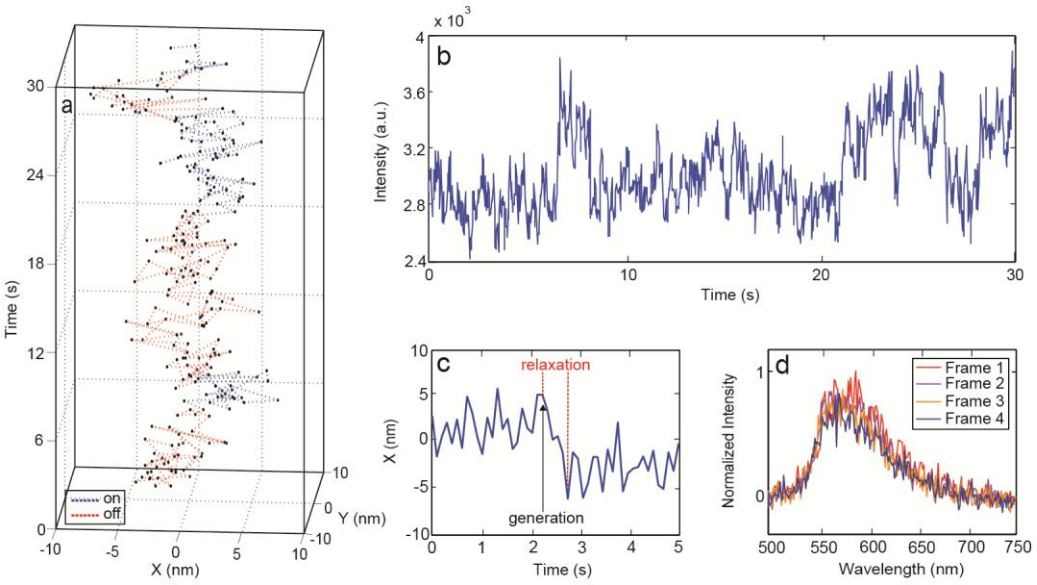

Supplementary Figure 8. Fluorescence intensity and centroid trajectories showing multiple cycles of polaron generation and recombination events. (a) The centroid position trajectory of a CPN, acquired at 50 Hz framerate. For plotting, every 5 points were binned together to reduce noise. Blue and red lines indicate that the corresponding intensity trajectory is at on or off state at the time, respectively. (b) The corresponding fluorescence intensity trajectory showing two-level blinking. (c) The centroid position

drifts after a polaron generation event. (d) Singe particle spectra during a polaron generation event. Frame 1 (red) corresponds to the spectrum right before the generation event. Frame 2 and 3 correspond to the spectrum during the centroid drift. Frame 4 (blue) corresponds to the spectrum after the drift.

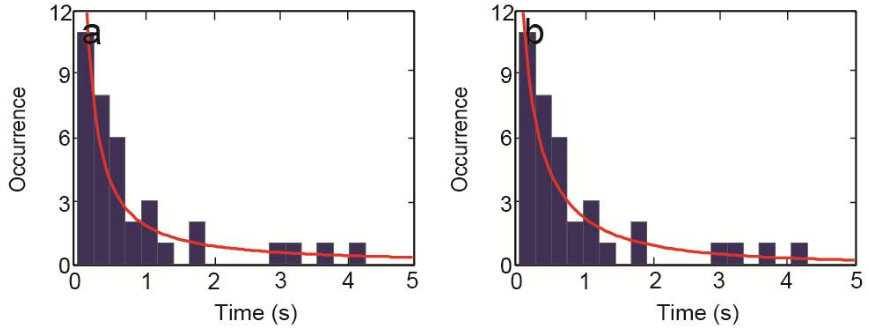

Supplementary Figure 9. Polaron hopping time distribution determined from change point analysis. The histogram is fit to (a) a power law function, $y = Ax^{-k}$, $k = 1.2$ (b) a stretched exponential function $y = Ae^{-(\frac{x}{\tau_{\mathrm{kww}}})^{\beta}}$, $\tau_{\mathrm{kww}} = 0.07\ s$, β= 0.45.

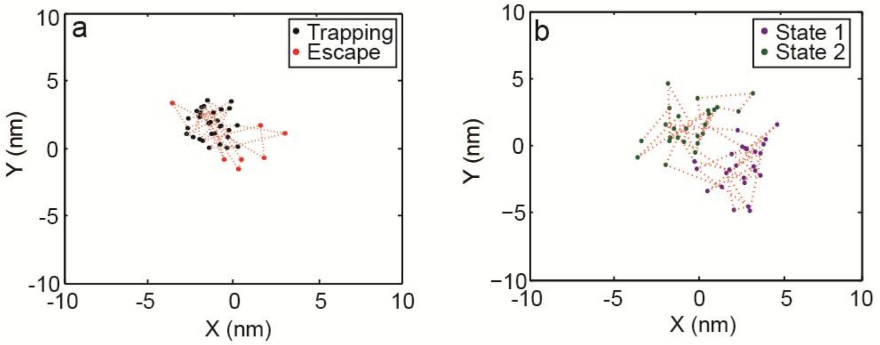

Supplementary Figure 10. Typical hopping behavior of polarons. (a) The scatter plot of a polaron position trajectory. According to the emission spectrum analysis, the scatter plot shows trapping (black dot) and escaping (red dot) dynamics of a polaron. (b) The scatter plot of a polaron position trajectory. Based on the emission spectrum analysis, the scatter plot can be separated into two trap states.

Supplementary Notes

Supplementary Note 1

Nanoparticle Characterization

F8BT nanoparticles were prepared using a nano-reprecipitation method described previously.[1,2] Freshly prepared F8BT CPNs were filtered using a cross-flow filtration column (pore size ~10 nm) to narrow the nanoparticle size distribution (Supplementary Fig. 1a). Before the filtration, the particle size determined from DLS measurement is 25.4±12.1 nm in diameter (Supplementary Fig. 1b). After the filtration, the particle size reduces to 11.2±3.7 nm in diameter (Supplementary Fig. 1b). The F8BT CPNs were dispersed on a glass coverslip and scanned with a multi-mode AFM. The particle height before and after the filtration are 22.2±10.2 nm and 8.9±3.1 nm, respectively (Supplementary Fig. 1d, f). These results indicate that most of the large nanoparticles were removed by the filtration. The UV−vis and fluorescence spectra of bulk F8BT CPNs suspension are broad and featureless with absorption and emission peaks around 450 nm and 550 nm (Supplementary Fig. 4a), which is consistent with previously reported values.[3] The single particle spectra of the filtered F8BT CPNs show particle to particle variations. Most of the CPNs exhibit emission spectra that are consistent with mixed phase F8BT (Supplementary Fig. 4b).[4] A small fraction of the CPNs exhibit blue emission with no red phase (Supplementary Fig. 4c), perhaps indicating a highly disordered conformation.

Supplementary Note 2

Determination of Tracking Uncertainty

In the polaron tracking experiment, we estimated the localization uncertainty of individual nanoparticles based on shot noise and the focal characteristics of the imaging setup, which is given by the following expression, [5]

$$\sigma = \sqrt{\frac{s^2}{N} + \frac{\alpha^2/12}{N} + \frac{8\pi s^4 b^2}{\alpha^2 N^2}} \quad (1)$$

where s is the standard deviation (STD) of the point spread function (PSF) and N is the number of detected photons used in the fitting, α is the pixel size, b is the background noise. STD of the single particle fluorescence spot is 130 nm. The pixel size of the setup is 66.5 nm (determined by imaging a calibration grid) and the background noise (due primarily to readout noise) is ~1.5. In this work, a transmission grating was placed in front of the detector to disperse single particle fluorescence. As a result, only a fraction of the total detected photons were used for single particle localization. Under 100 W per cm^2 excitation power density, 50 Hz framerate, 1-3 $\times 10^3$ photons were detected at the zeroth order (undiffracted) spot, resulting in a localization precision

of 2.5-3.8 nm per frame, according to supplementary equation 1. Autocorrelation was performed on raw trajectories to estimate the time constants of the dynamics. In case of highly trapped polaron with a hopping time constant much larger than the temporal resolution, we can bin frames together accordingly to reduce the localization uncertainty. The tracking uncertainty after binning is given by the expression $\sigma/\sqrt{n}$ (n is the number of frames binned together).

Supplementary Note 3

Single Particle Statistics

We conducted single particle fluorescence study of hundreds of F8BT CPNs. For CPNs showing two-level blinking behavior (multiple polaron dynamics complicate the fluorescence centroid analysis), we constructed particle histograms of average spectral drift (Supplementary Fig. 3a) and hole-polaron displacement (Supplementary Fig. 3b) associated with emission on/off transitions as well as histograms of average spectral drift (Supplementary Fig. 3c) and hole-polaron displacement (Supplementary Fig. 3d) associated with hole hopping events. As shown in Supplementary Fig. 3a, the spectral drifts associated with emission on/off transitions range from 0 nm to 20 nm with most occurrences around 8 nm. There are a small fraction of CPNs that exhibit minimal spectral drift over time, which corresponds to the blue-emitting CPNs, as discussed in the previous section. Of all the on/off transitions studied, 39% show discrete jumps and 61% show continuous drift/multiple-step hops in the fluorescence centroid. The hole polaron displacement associated with emission on/off transitions are typically <10 nm with most occurrences around 4 nm, consistent with the size of the nanoparticles (Supplementary Fig. 3b). As shown in Supplementary Fig. 3c, the spectral drift associated with polaron hopping events show most occurrences of less than 2 nm, which is consistent with polaron hopping between the red-emitting sites. The distribution of hole-polaron displacement associated with polaron hopping events (Supplementary Fig. 3d) is similar to the distribution of hole-polaron displacement associated with on/off transitions (Supplementary Fig. 3b), consistent with hole polarons relaxing to/hopping between red-emitting sites randomly distributed in CPNs (here we are only considering motion in the XY plane due to limitations of the experiment). In Supplementary Fig. 3e and f, we show histograms of hole polaron quenching depths in mixed phase CPNs and blue phase CPNs. It was observed that polarons generated in the red-emitting areas resulted in significantly higher quenching depths than polarons generated in the blue-emitting phase, indicating highly efficient exciton migration to quenchers in the red-emitting regions.

Supplementary Note 4

Hole Polaron Generation Processes

As the phenomena examined in this paper involve likely photogenerated hole polarons in nanoparticles of F8BT polymer, here we discuss some relevant aspects of the charge generation processes in conjugated polymers and in the context of the F8BT nanoparticles. In neat conjugated polymers, a delocalized state above the S1 excited state is thought to be associated with charge generation.[6-8] In this state, the hole is confined while the electron can extend to the neighboring units, which facilitates charge separation.[6] Excess photon/thermal energy or exciton-exciton annihilation processes can provide additional energy for excitons to access the delocalized state and lead to exciton dissociation.[7,9-11] There are also reports of charge carrier generation in neat conjugated polymers even in conditions of little excess energy.[12-14] Under these circumstances, there is evidence that grain boundaries act as exciton dissociation sites, which perhaps is related to trapping of one of the electron-hole pair at the interface and delocalizing of the other one in the crystalline domain.[14-16] When conjugated polymer is doped with electron acceptors, charge generation is facilitated by the energy level offset between the donor and the acceptor. For the charge-transfer state at the donor/acceptor interface, there is also discussion regarding the potential role of a higher-lying delocalized state in the process of charge separation.[8,17] Recently, ultrafast pump-push experiments have shown that the higher-lying delocalized state can be readily accessed using a visible pump pulse followed by an IR push pulse.[8] The delocalized state is short-lived, indicating electron-hole pair dissociation within 1 ps. Simulation has shown that charge carriers are delocalized on the fs timescale and then are stabilized (trapped) by the geometric relaxation of the polymer chain.[8] In our experiment, polaron generation could occur through direct exciton dissociation or could involve charge separation at a donor/acceptor interface or at a grain or domain boundary. Potential electron acceptors include molecular oxygen, chemical defects with high electron affinity (for example, carbonyl defects) and molecules/electron deficiencies on the silanized glass substrate.[18-20] It should be noted that none of the charge generation pathways discussed here is expected to be efficient in undoped F8BT nanoparticles, which is consistent with the low charge carrier yield in neat F8BT as well as our observation that there is typically a single quencher or none presented in a nanoparticle at a given time under the low to moderate excitation conditions of the experiment. Since exciton dissociation is typically characterized as occurring on ultrafast timescales well below the time resolution of the present experiment, in this paper we are principally concerned with the motion of hole polarons (after charge separation) as well as the polaron and exciton energy landscape.

Supplementary Note 5

Single Particle Fluorescence Intensity and Spectrum Centroid Trajectories

As discussed earlier, in the single particle spectrum study, it was observed that a small fraction of the CPNs (~5%) exhibit blue emission with no red phase. The fluorescence intensity trajectories of these CPNs show clear fluctuations over time. Autocorrelation analysis of the fluorescence intensity trajectories indicates that the timescale of the intensity fluctuation is around several

seconds, which is in range with the timescale of polaron generation and recombination dynamics. It is likely that there were polarons generated in these CPNs. However, due to the fact that there is no red emitting phase, generation/recombination of quenchers doesn't result in a pronounced spectral shift. An example is given in Supplementary Fig. 5a, b, the spectrum centroid trajectory only exhibits minimal fluctuations over time.

In some mixed phase F8BT CPNs, we observed multiple level intensity fluctuations likely due to generation and recombination dynamics of multiple polarons. The spectrum centroid trajectories of these CPNs look very similar to the corresponding fluorescence intensity trajectories (Supplementary Fig. 5c, d). Generation of quencher almost always shifts the spectrum centroid to a lower wavelength. This phenomenon is consistent with the initial fluorescence intensity decay behavior discussed in the main text that the hole polarons tend to selectively occupy red emitting sites in CPNs.

Supplementary Note 6

Polaron Relaxation Process

In some trajectories, the polaron relaxation process is complex that some intermediate states can be observed before the polaron was finally captured by a trap site. An example is given in Supplementary Fig. 7. When the hole polaron was generated, the fluorescence centroid position exhibited a continuous drift and the spectrum centroid shifted to a lower wavelength, indicating that the hole polaron initially moved to a red-emitting area. The polaron only stayed there briefly and then hopped into a blue-emitting area, indicated by a red shift in the spectrum centroid. At the same time, the fluorescence centroid position also exhibited abrupt jumps (highlighted by the red bands in Supplementary Fig. 7). In the end, the spectrum centroid and fluorescence centroid position stabilized. As indicated by the spectrum and fluorescence centroid, the hole polaron was captured by a different red-emitting trap site (probably a deep trap, as suggested by the long trapping time of the polaron).

In the main text, we mentioned that, for some CPNs, during different generation and recombination cycles, the fluorescence centroid tends to drift to the same location. An example is given in Supplementary Fig. 8a. The red and blue lines indicate the fluorescence centroid positions during intensity off and on segments, respectively. During different generation events, the fluorescence centroid undergoes continuous drifts and stabilizes at about the same location (Supplementary Fig. 8a, c), indicating that the polaron was captured by the same trap site. After the fluorescence centroid stabilized, we observed that the red shoulder of the single particle spectrum was quenched, indicating that the hole polaron was relaxed to a red-emitting region (Supplementary Fig. 8d).

Supplementary Note 7

Representative Polaron Hopping Trajectories

For dozens of polaron position trajectories analyzed, the polaron hopping behavior can be roughly grouped into two categories. When the trap density is low in a CPN, trapping/escaping dynamics is most commonly observed, i.e., hole polaron occasionally escapes from the trap and then is quickly recaptured by the same trap (Supplementary Fig. 10a). The polaron position trajectory exhibits minimal movements with occasional spikes indicating polaron escaping from the trap. In other cases, the polaron position trajectories exhibit frequent abrupt movements (Supplementary Fig. 10b). Depending on trap density, the hole polaron either exhibits a random walk-like behavior, which involves visiting many sites, or shows repeated hopping between a small number of sites. The positions of the traps were located using position histograms/scatter plots and used to construct a nanoscale map inside the CPN.

Supplementary Methods

Calibration and Determination of Single Particle Emission Spectrum

To measure single particle fluorescence spectra, a transmission grating with 300 grooves per mm was placed between the 500 nm long-pass filter and the sCMOS camera to disperse the fluorescence emission from CPNs. Gaussian-shaped PSFs of single particles can be observed at the $n = 0$ spot while long stripes, which correspond to the fluorescence spectra of individual CPNs, can be observed at the $n = 1$ spot (Supplementary Fig. 2c, d). The bright stripe of each CPN was summed along the Y axis (15 pixels in total) to obtain the single particle emission spectrum. To calibrate the spectra, we replaced the 500 nm long-pass filter with a 540 ± 10 nm band-pass filter. We determined that the center of the $n = 0$ and $n = 1$ spots were separated by 773 pixels (Supplementary Fig. 2a, b). By dividing 450 nm with 773 pixels, we obtained a dispersion factor of 0.7 nm per pixel. Based on the single particle fluorescence spot width of roughly 4-5 pixels (FWHM), which roughly corresponds to the slit width of the spectrometer, the spectroscopic resolution is roughly 3.5 nm.

Change Point Analysis using Haar Wavelet

In this work, we used change point detection to localize many different types of dynamics, including fluorescence blinking dynamics, spectrum centroid shifts and polaron hopping dynamics. The change point analysis employed is based on Haar wavelet transform. Haar wavelet is typically used to detect abrupt changes above small, fast fluctuations. Local maxima in Haar wavelet transform coefficient indicate change points in a time series. The wavelet transform coefficient is calculated by,

$$C(t_0, a) = \frac{1}{a}\int_R S(t)\psi(\frac{t-t_0}{a})dt \quad (2)$$

where $S(t)$ is a time series, ψ is a wavelet function has width a, centered at t_0. a is scaling of wavelet transform, which can be considered as bin size. For the different dynamics mentioned above, the procedure for change point analysis is similar. Here, we will use polaron hopping dynamics as an example. The original polaron position trajectory is given in Supplementary Fig. 6a. In this trajectory, polaron hops between two sites and exhibits different lifetime at each site. wavelet transform was first performed at scale 2 to estimate the global noise level (Supplementary Fig. 6b). The median absolute deviation of the wavelet transform coefficients at scale 2 was used as a threshold to differentiate real transitions from noise.[21] As the scale increases, the hopping events were separated from noise, indicated by peaks above the threshold (Supplementary Fig. 6c). The forward and backward hopping events are indicated by the positive and negative peaks in the wavelet transform coefficients. From the separations of the peaks in the wavelet transform coefficients, we can determine the hopping time distribution of the polaron. Typically, good separation between real transitions from noise can be achieved for a range of scales. Eventually, when the scale becomes too large, i.e., the size of the wavelet becomes bigger than the separation of the transition events, multiple hopping events merge together in the wavelet transform coefficients (Supplementary Fig. 6d). As a result, the hopping time distribution determined is no longer accurate. Therefore, it is important to perform wavelet analysis on multiple scales and compare the results for consistency. In addition, the average hopping time determined from the wavelet analysis is compared to the time constant from autocorrelation analysis to ensure consistency. The hopping time distribution determined can be fit to a power law or a stretched-exponential function (Supplementary Fig. 9), consistent with dispersive charge transport occurring over a range of time scales.

Supplementary References

1 Wu, C., Szymanski, C. & McNeill, J. Preparation and encapsulation of highly fluorescent conjugated polymer nanoparticles. *Langmuir* **22**, 2956-2960 (2006).

2 Tian, Z., Yu, J., Wu, C., Szymanski, C. & McNeill, J. Amplified energy transfer in conjugated polymer nanoparticle tags and sensors. *Nanoscale* **2**, 1999-2011 (2010).

3 Wu, C., Bull, B., Szymanski, C., Christensen, K. & McNeill, J. Multicolor conjugated polymer dots for biological fluorescence imaging. *ACS nano* **2**, 2415-2423 (2008).

4 Grey, J. K. *et al.* Effect of temperature and chain length on the bimodal emission properties of single polyfluorene copolymer molecules. *J. Phys. Chem. B* **110**, 18898-18903 (2006).

5 Thompson, R. E., Larson, D. R. & Webb, W. W. Precise nanometer localization analysis for individual fluorescent probes. *Biophys. J.* **82**, 2775-2783 (2002).

6 Köhler, A. *et al.* Charge separation in localized and delocalized electronic states in polymeric semiconductors. *Nature* **392**, 903 (1998).

7 Stevens, M. A., Silva, C., Russell, D. M. & Friend, R. H. Exciton dissociation mechanisms in the polymeric semiconductors poly (9, 9-dioctylfluorene) and poly (9, 9-dioctylfluorene-co-benzothiadiazole). *Phys. Rev. B* **63**, 165213 (2001).
8 Bakulin, A. A. *et al.* The role of driving energy and delocalized states for charge separation in organic semiconductors. *Science* **335**, 1340-1344 (2012).
9 Martini, I. B., Smith, A. D. & Schwartz, B. J. Exciton-exciton annihilation and the production of interchain species in conjugated polymer films: comparing the ultrafast stimulated emission and photoluminescence dynamics of MEH-PPV. *Phys. Rev. B* **69**, 035204 (2004).
10 Basko, D. & Conwell, E. Hot exciton dissociation in conjugated polymers. *Phys. Rev. B* **66**, 155210 (2002).
11 Grancini, G. *et al.* Hot exciton dissociation in polymer solar cells. *Nat. Mater.* **12**, 29-33 (2013).
12 Dicker, G., de Haas, M. P., Siebbeles, L. D. & Warman, J. M. Electrodeless time-resolved microwave conductivity study of charge-carrier photogeneration in regioregular poly (3-hexylthiophene) thin films. *Phys. Rev. B* **70**, 045203 (2004).
13 Zaushitsyn, Y. *et al.* Ultrafast light-induced charge pair formation dynamics in poly [3-(2′-methoxy-5′ octylphenyl) thiophene]. *Phys. Rev. B* **70**, 075202 (2004).
14 Reid, O. G. *et al.* The influence of solid-state microstructure on the origin and yield of long-lived photogenerated charge in neat semiconducting polymers. *J. Polym. Sci., Part B: Polym. Phys.* **50**, 27-37 (2012).
15 Reid, O. G., Pensack, R. D., Song, Y., Scholes, G. D. & Rumbles, G. Charge photogeneration in neat conjugated polymers. *Chem. Mater.* **26**, 561-575 (2013).
16 Paquin, F. *et al.* Charge separation in semicrystalline polymeric semiconductors by photoexcitation: is the mechanism intrinsic or extrinsic? *Phys. Rev. Lett.* **106**, 197401 (2011).
17 Bakulin, A. A., Silva, C. & Vella, E. Ultrafast spectroscopy with photocurrent detection: watching excitonic optoelectronic systems at work. *J. Phys. Chem. Lett.* **7**, 250-258 (2016).
18 List, E. *et al.* Charged defects in highly emissive organic wide-band-gap semiconductors. *Appl. Phys. Lett.* **76**, 2083-2085 (2000).
19 Yan, M., Rothberg, L., Papadimitrakopoulos, F., Galvin, M. & Miller, T. Defect quenching of conjugated polymer luminescence. *Phys. Rev. Lett.* **73**, 744 (1994).
20 Seemann, A. *et al.* Reversible and irreversible degradation of organic solar cell performance by oxygen. *Solar Energy* **85**, 1238-1249 (2011).
21 Chen, K., Wang, B., Guan, J. & Granick, S. Diagnosing heterogeneous dynamics in single-molecule/particle trajectories with multiscale wavelets. *ACS nano* **7**, 8634-8644 (2013).
